# Contribution of an Aged Microenvironment to Aging-Associated Myeloproliferative Disease

**DOI:** 10.1371/journal.pone.0031523

**Published:** 2012-02-21

**Authors:** Virag Vas, Corinna Wandhoff, Karin Dörr, Anja Niebel, Hartmut Geiger

**Affiliations:** 1 Department of Dermatology and Allergic Diseases, University of Ulm, Ulm, Germany; 2 Division of Experimental Hematology and Cancer Biology, Cincinnati Children's Hospital Medical Center, University of Cincinnati, Cincinnati, Ohio, United States of America; Emory University, United States of America

## Abstract

The molecular and cellular mechanisms of the age-associated increase in the incidence of acute myeloid leukemia (AML) remain poorly understood. Multiple studies support that the bone marrow (BM) microenvironment has an important influence on leukemia progression. Given that the BM niche itself undergoes extensive functional changes during lifetime, we hypothesized that one mechanism for the age-associated increase in leukemia incidence might be that an aged niche promotes leukemia progression. The most frequent genetic alteration in AML is the t(8;21) translocation, resulting in the expression of the AML1-ETO fusion protein. Expression of the fusion protein in hematopoietic cells results in mice in a myeloproliferative disorder. Testing the role of the age of the niche on leukemia progression, we performed both transplantation and *in vitro* co-culture experiments. Aged animals transplanted with AML1-ETO positive HSCs presented with a significant increase in the frequency of AML-ETO positive early progenitor cells in BM as well as an increased immature myeloid cell load in blood compared to young recipients. These findings suggest that an aged BM microenvironment allows a relative better expansion of pre-leukemic stem and immature myeloid cells and thus imply that the aged microenvironment plays a role in the elevated incidence of age-associated leukemia.

## Introduction

Acute myeloid leukemia is rarely diagnosed before the age of 40, with a subsequent exponential increase in the incidence. AML is thus an aging-associated cancer/leukemia. The molecular and cellular mechanisms of the age-associated increase in leukemia remains poorly understood. It is believed that AML is driven by leukemia initiating cells (LICs) which are similar in nature to hematopoietic stem cells (HSCs) [1]–[3].

An accepted paradigm is that both cell intrinsic as well as extrinsic factors contribute to leukemia formation and its progression [4]. The cell intrinsic mutation theory for example suggests that at least two types of ‘collaborative’ hits are required to cause leukemia: one that confers a survival advantage for the cell (affecting for example a kinase gene), and a second defect resulting in a block in hematopoietic differentiation (exemplified by core binding factor) [5]. It is also widely accepted that during in leukemia, leukemic stem cells lodge into normal stem cell niches in the BM and initiate a crosstalk with their surrounding tissue, which might result initiation of the disease, repression of normal HSC functions, alter the lineage differentiation of leukemic cells or regulate the response to drugs [6]–[11].

Aging in hematopoiesis manifests itself as decreased immune response, increased contribution to the myeloid cell lineage, late-onset anemia and reduced regenerative capacity of stem cells and is driven by cell intrinsic and extrinsic factors [12]–[14]. Ineffective lymphoid cell production from aged HSCs has been for example described as a primarily cell-intrinsic aging-associated change [15]. Novel data further suggest that aging changes the clonal composition of the HSC compartment because of a relative expansion of myeloid-biased HSCs (clonal diversity/expansion model) [16], [17]. Furthermore, the BM microenvironment itself changes with age as indicated by decreased bone formation, enhanced adipogenesis and changes in extracellular matrix (ECM) components [18]–[20].

Recent analyses shed some light on the likely contribution of hematopoietic cell intrinsic factors to age-associated leukemia [21]. Signer et al. for example showed that age-associated HSC intrinsic skewing towards myelopoiesis might play a causative role for the myeloid dominance of leukemias in elderly [22]. Other data indicate that aged HSCs show increased levels of γH2AX staining, a surrogate marker for DNA double strand breaks [23] and that young HSCs might be able to accumulate cytogenetical aberrations over a lifetime as a result of incorrectly repaired DNA damage [24]. In contrast, arguing against solely cell intrinsic mechanisms in age-associated increase in the incidence of leukemia is for example the finding that aged B-lymphoid progenitors allow for a greater competitive advantage of leukemic cells compared to young B-lymphoid progenitors and that this decline in competitiveness of aged B-lymphoid progenitors increased the progression of bcr-abl driven leukemia [25].

It is thus likely that both cell-extrinsic and -intrinsic effects contribute to the age-associated increase in the incidence of leukemias like AML. The role though of the aged niche/microenvironment on leukemia initiation or progression has not been analyzed in great detail so far. A testable hypothesis is that an aged microenvironment, in comparison to a young one, preferentially facilitates leukemia progression due to better selection of the fittest and thus most leukemic cells. This could result in a faster transition time from pre-leukemia to leukemia in an aged BM microenvironment, translating into an increased incidence of leukemia with age.

The translocation (8∶21) is a frequent genetic abnormality associated with AML and results in expression of the AML1-ETO fusion protein. It represses genes necessary for myeloid and lymphoid development and by this means inhibits multiple differentiation pathways primarily in HSCs [26]. The presence of the AML1-ETO fusion protein in HSCs alone does not cause leukemia, as secondary genetic lesions ((like ICSBP deficiency [27], mutation in FLT3 [28] and C-KIT [29]) and/or potential leukemia supporting niche-effects are necessary for the progression to AML. This murine AML-ETO model is therefore an established model of a myeloproliferative disorder and a potential pre-leukemic disease thought to arise from LIC derived from HSCs [30], [31].

To investigate the role of an aged microenvironment on leukemia progression, we compared the influence of young and aged stroma/microenvironment on pre-leukemic HSC expansion *in vitro* and *in vivo* and on disease progression in AML-ETO induced myeloid proliferation. Our data demonstrate that *in vivo*, AML-ETO positive, myeloproliferation-initiating Lin−, Sca-1+, C-kit+ (LSK) cells expand better in an aged BM niche/environment compared to a young one. We therefore conclude that the age of the niche is able to influence the size of the pool of myeloproliferation-initiating cells, which might be one factor contributing to the increased incidence of AML with age.

## Results

### Elevated AML-ETO+ Myeloid Cell Production in an Aged Niche/Microenvironment *in vivo*

**Figure 1:**
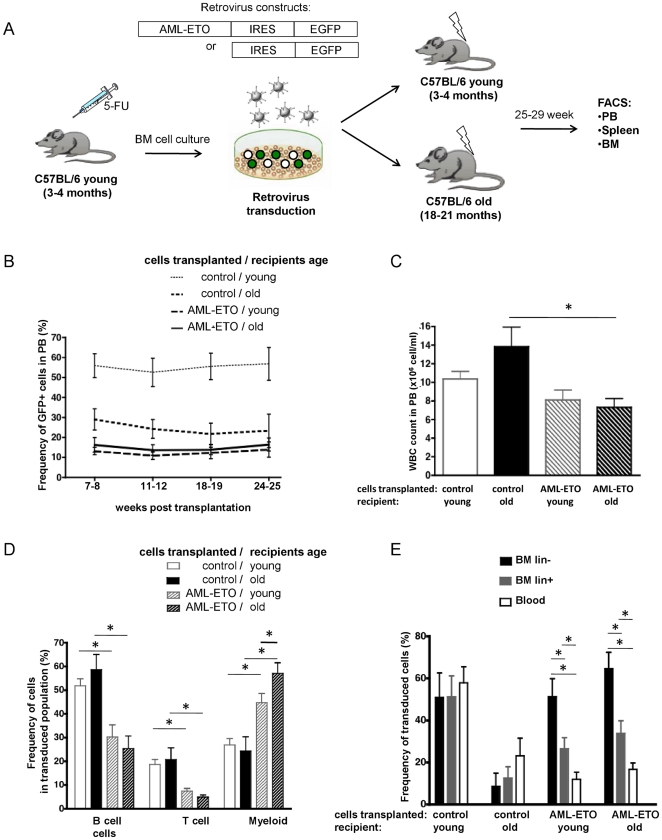
Elevated AML-ETO+ myeloid cell production in aged recipients. (**A**) Experimental setup. (**B**) Percentage of GFP positive cells in PB in young and aged mice transplanted with control or AML-ETO positive BM cells (**C**) White blood cell count (WBC) at 25–29 week post transplantation at termination of the experiment. (**D**) Lineage distribution (T-, B- and myeloid cells) of the transduced cell populations in PB of young and aged recipients. (**E**) Percentage of GFP positive cells among un-differentiated lineage marker negative (lin−) and differentiated (lin+) cells in BM and among cells in PB of young and aged recipient mice. (Control-cell transplanted in young recipient n = 8, control-cell transplanted in old recipient n = 5, AML-ETO+cell transplanted in young recipient n = 12, AML-ETO+cell transplanted in old recipient n = 11, from a total of 3 independent biological repeats, * = p<0.05). Bars represent the mean ± SEM.

To test the hypothesis that the age of the niche influences the progression of pre-leukemia *in vivo*, we transplanted young AML-ETO transduced BM cells in either aged or young mice (Figure 1A), exposing cells to a young or an aged BM microenvironment. While, as anticipated by reports published by Liang et al. [32] that indicate reduced homing in an aged microenvironment, the frequency of GFP+ cells in PB was lower in aged recipients transplanted with control cells compared to young recipients, animals receiving the AML-ETO+ graft presented with the lowest chimerism in PB. Reconstitution though remained stable for the duration (up to 25 weeks) of the experiment in both age groups (Figure 1B). The white blood cell count in the PB was significantly lower in old mice transplanted with AML-ETO+ cells compared to the old control mice (Figure 1C). The PB analysis showed decreased (B220+) B cell and (CD3+) T cell but increased myeloid cell (Mac1+ and Gr1+ cells combined) production among the AML-ETO-transduced cells compared to the control-GFP+ cells at every time point analyzed (Figure 1D and data not shown). These results confirm that in both young and aged recipients the differentiation potential of AML-ETO+ cells was disturbed and skewed towards myelopoiesis. Remarkably, AML-ETO expressing cells produced more myeloid cells in old compared to young recipients (Figure 1D), supporting our initial hypothesis of a differential action of young and aged microenvironment on pre-leukemic stem cells.

Further analyses revealed that, in contrast to control cells transplanted into young and aged recipients, the contribution of AML-ETO transduced cells to un-differentiated BM cells (Lin−) population was elevated compared to differentiated cells (Lin+) in BM and even further elevated compared to the chimerism reported for PB (Figure 1E). While this maturational arrest in the early stages of hematopoiesis in AML-ETO transduced cells was similar in young and aged recipients, it differed between old recipients receiving control versus AML-ETO+ BM cells. This resulted an inversion of the differentiation arrest pattern seen in control cells in aged recipients compared to AML-ETO+ cells in aged recipients, demonstrating a strong and dominant influence of AML-ETO on cell differentiation programs.

### Elevated Immature Myeloid Cell Load in Aged Recipients

**Figure 2:**
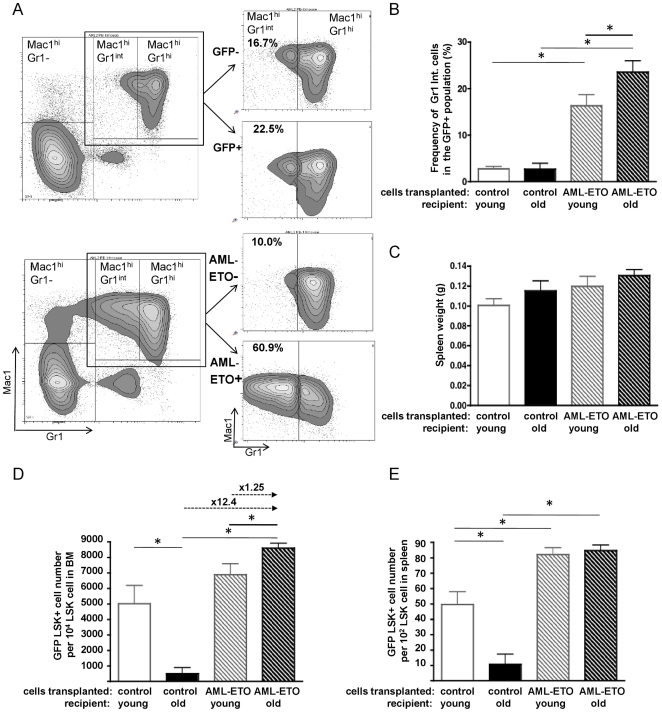
Elevated immature myeloid cell load and expansion of myeloproliferation-initiating stem cells in an aged microenvironment. (**A**) Representative FACS profiles of PB from an aged control-transplanted (upper panel) and an aged AML-ETO-transplanted (lower panel) recipient at 25 weeks post transplantation for determination of immature myeloid cells. Immature myeloid cells were identified as Gr1^int^Mac1^hi^ cells whereas Gr1^hi^Mac1^hi^ cells represent mature myeloid cells (right panels: highlighting the Gr1Mac1 double positive population divided into GFP+ and GFP− cell-fraction and Gr1^int^Mac1^hi^ and Gr1^hi^Mac1^hi^ subpopulations). (**B**) Frequency of immature myeloid cells (Gr1^int^Mac1^hi^) among GFP+ cells in PB at 24–25 weeks post transplantation. (**C**) Spleen weight in the recipient animals. (**D**) Quantitation of flow cytometric analyses of BM derived GFP+LSK cells in recipient animals. The relative increase of GFP+LSK cells among the experimental groups is depicted on top of the arrows. (**E**) Quantitation of flow cytometric analyses of spleen derived GFP+LSK cells in recipient animals. (Control-cell transplanted in young recipient n = 8, control-cell transplanted in old recipient n = 5, AML-ETO+cell transplanted in young recipient n = 12, AML-ETO+cell transplanted in old recipient n = 11, from a total of 3 independent biological repeats, * = p<0.05). Bars represent the mean ± SEM.

A widely accepted hallmark of the pre-leukemia phase of AML in the murine model is an increase in the frequency of immature myeloid cells in PB. We quantified these not terminally differentiated myeloid cells by flow cytometry in the PB samples according to standard protocols [27], [30]. Cells expressing Gr1 at intermediate levels (Gr1^int^) represent immature myeloid forms and were, as anticipated, present at a low frequency (approx. 10–20%) in the Mac1+Gr1+ myeloid cell population of control mice as well as in the un-transduced (AML-ETO−) cell population in mice transplanted with AML-ETO+ cells, while they are found at high frequency (approx. 60%) among AML-ETO transduced myeloid cells, consistent with an overall enlarged immature myeloid compartment (Figure 2A) and myeloproliferative phenotype [33]. Determining differences among experimental groups, our data demonstrated a higher frequency of the (Gr1^int^, Mac1^hi^) immature myeloid cells within AML-ETO+ cells in old compared to young recipient mice, resulting in an elevated immature myeloid cell load in aged recipients (Figure 2B). Overt leukemia frequently results in an enlarged spleen. Consistent with a halt of AML-ETO+ cells in a pre-leukemic state in our mouse model, spleen weights were not significantly elevated in mice transplanted with AML-ETO transduced cells and similar in young and old animals (Figure 2C). In summary, our analyses indicate that in the AML1-ETO model an aged microenvironment does not facilitate a pre-leukemia to leukemia conversion but rather allows for an expansion of immature myeloid cells in PB.

### Expansion of Myeloproliferation-Initiating Stem Cells is Supported by an Aged Niche

A rare cell population within BM with the surface phenotype Lin−, Sca-1+, c-Kit+ (LSK) is enriched for primitive hematopoietic stem and progenitor cells, and at the same time has been shown in the murine AML-ETO pre-leukemia model to be highly enriched for myeloproliferation-initiating, transplantable pre-leukemia stem cells [30]. To subsequently determine the frequency of primitive hematopoietic cells as well as these myeloproliferation-initiating stem cells in our transplantation experiments, the LSK cell population was analyzed both in BM and spleen. Transplanted young and aged mice were sacrificed 25–29 weeks post transplantation, when aged mice were reaching their likely maximal lifespan of 25–27 months. As old and young recipients were transplanted with the same pool of transduced BM cells, any difference in the LSK population between the groups could be assigned to the age of the recipient. Consistent with chimerism data already presented in Figure 1E, aged animals transplanted with cells transduced with control vector demonstrated a very low level of GFP+ chimerism in the LSK population compared to young recipients, indicating that an aged niche limits the expansion of transduced LSK cells. In contrast, the frequency of GFP expressing cells among the LSK population was significantly elevated in aged recipients transplanted with AML-ETO transduced cells compared to aged animals transplanted with control cells, and significantly elevated compared to young animals transplanted with AML-ETO+ cells (Figure 2D). The AML-ETO+ LSK cell-pool in aged recipients was on average 1.25 fold increased compared to young recipients and 12.4 fold compared to the aged control mice.

To determine whether the increase in the number of AML-ETO+ LSK cells in aged recipients was due to local BM niche or rather a systemic effect affecting multiple hematopoietic organs, we subsequently determined the GFP expression in the LSK compartment in another hematopoietic organ, the spleen. Similar to the BM analyses, the GFP+LSK frequency was low in spleens of aged recipients transplanted with control cells compared to the frequency in young recipients (Figure 2E). In contrast to the BM though, the relative number of AML-ETO+ LSK cells in the spleen was similar in aged and young recipients transplanted with AML-ETO+ hematopoietic cells, indicating that not systemic, but local niche/environment factors inside the BM are responsible for the age-associated higher level of expansion of myeloproliferation-initiating LSK cells in the BM of aged animals.

### *In Vitro* Culture of AML1-ETO+ Stem/Progenitor Cells on Aged and Young Endosteal Cells

**Figure 3:**
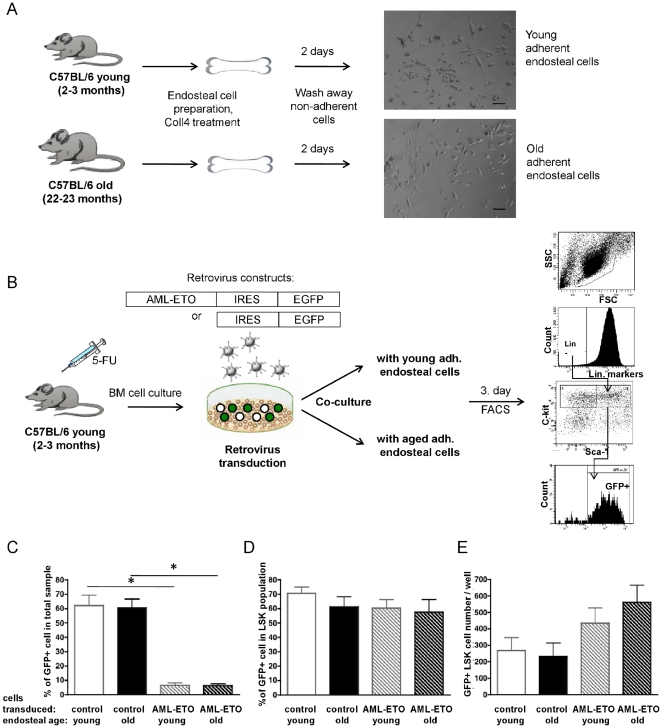
*In vitro* culture of AML1-ETO positive stem/progenitor cells on aged and young endosteal cells. (**A**) Experimental set-up for isolation of a cell fraction close to the endosteum ( =  endosteal cells) and representative phase contrast images (×10) from adherent endosteal cells from young and aged mice, bar represent 50 µm (**B**) Experimental set-up for co-culture experiments and FACS analysis for analysis of the Lin−,Sca-1+, c-Kit+ (LSK) cell compartment. (**C**) Frequency of GFP+ cells among all cells, (**D**) frequency of GFP+ cells in the LSK population (**E**) and the total number GFP+LSK cells. n = 3, * = p<0.05. Bars represent the mean ± SEM.

HSCs as well as LICs interact with a variety of stromal cell types in their BM microenvironment, such as endothelial cells, reticular cells, osteoblasts and mesenchymal stem cells, forming a niche [1], [34]. We developed an short-term *in vitro* cell co-culture assay to determine the influence of the age of stroma cells on the expansion of AML-ETO+ myeloproliferation-initiating LSK cells *in vitro* [26], [35] to determine whether this 2D *in vitro* co-culture assay might be able to re-capitulate the increase in AML-ETO+ LSK cells in an aged microenvironment, which would then allow to further determine cellular and molecular mechanisms. For the preparation of the stroma cell population, long bones isolated from young and aged mice and in which the bone marrow was flushed out were treated with Collagenase IV (Figure 3A) to isolate cells close to the endosteum. This cell fraction has been described as a source of osteoblasts [36] and MSC [37], thus including a high complexity of cell types resembling an endosteal microenvironment. This population was kept without any further passage *in vitro* to avoid selection for distinct cell sub-types. To initiate the co-culture experiments, BM cells from young 5-FU treated mice were transduced with control or AML-ETO retrovirus and identical numbers of cells seeded on top of the adherent old or young endosteal cells (Figure 3B). The total number of LSK cells, a population representing early hematopoietic progenitor cells also containing the majority of myeloproliferation-initiating cells [30], [31] were determined on day 3 of co-culture before stem cells started to differentiate. While the contribution of AML-ETO transduced cells was lower compared to control transduced cells in the overall cell pool, (Figure 3C) the frequency of transduced cells within the LSK population was similar for both AML-ETO and control vector, indicating that the AML-ETO vector efficiently transduces stem cells (Figure 3D). The total number of AML-ETO+ LSK cells showed a slight trend for elevated numbers when short term cultured on aged endosteal cells compared to young cells, but remained overall relatively similar in response to incubation on young and aged endosteal cells (Figure 3E). The short-term 2D co-cultivation of pre-leukemic stem cells and endosteal cells does not re-capitulate the *in vivo* measured aged-stromal support in respect to AML-ETO positive LSK cells.

## Discussion

We compared the influence of a young and aged stroma/microenvironment on proliferation and progression of AML-ETO driven myeloproliferation in mice, both *in vitro* and *in vivo*. The number of AML-ETO+ LSK cells, representing myeloproliferation-initiating cells, was significantly elevated in aged mice transplanted with young AML-ETO transduced BM cells compared to young recipients transplanted with the identical pool of cells. This observation was specific to the BM microenvironment and not found in spleen, suggesting that this phenotype is regulated by the local niche/microenvironment in BM and not by systemic influences. These results further imply a correlation between the age of the stroma/microenvironment and the relative increase of the size of the pool of myeloproliferation-initiating stem cells and the frequency of immature myeloid cells in PB and are in line with a previously reported clinical study observing a significantly higher number among elderly AML patients showing immature (CD34+/CD33+) blast cell population [38], [39].

Our data can be explained by both better support of the aged BM microenvironment of the pre-leukemic stem/progenitor cells or a stronger suppressive effect of a young stroma. This includes the possibility that AML-ETO+ cells might be able to modify the aged BM microenvironment more effectively to create tumor supportive niches compared to a young, in which case aged niches would initially not present with a direct supportive property but would acquire this ability in the presence of AML-ETO+ cells. Our data with respect to the expansion of LSK cells transduced with a control vector (Fig. 3D) suggest that in general a young microenvironment is not suppressive, rendering it more likely that an aged microenvironment either directly or indirectly supports the expansion of pre-leukemic stem cells. Niche involvement in leukemic cell fate determination – together with intrinsic changes in HSCs and LICs with age – thus form an emerging concept in understanding mechanisms of the age-associated increased incidence of leukemia. The major contributing factors in the BM niche and mechanisms causing the expansion of pre-leukemic cell populations need to be further determined.

A diverse set of cells located close to the endosteum was used in our *in vitro* cell co-culture experiments to mimic an endosteal niche/microenvironment. In contrast to the *in vivo* experiments, AML-ETO+ LSK cells when cultivated with aged compared to young stroma did not show an enhanced expansion in our assays (Figure 3D). Whether an aged stroma supports greater myeloid expansion or differentiation needs to be determined in additional experiments. The transient and as well as simple nature of the co-culture assays in such a reductionist approach and as performed in our laboratory is thus not suitable to further dissect cellular or molecular mechanisms involved in expansion of AML-ETO+ LSK cells in an aged micro-environment. Novel experiments will thus be necessary to investigate whether other co-culture protocols will be better suitable to identify regulatory mechanisms of the aged micro-environment. Our co-culture assays lacked for example the long-term effect of the influence of the stroma cells as the experiment lasted only one to two stem cell division cycles (3 days to avoid differentiation) as well as the three-dimensional complex structure of the BM milieu. Although our set-up aimed at including the most divers types of endosteal stroma cells, another possibility might be that our endosteal cell preparations did not contain the cell type (or types) necessary for enhanced expansion of AML-ETO+LSK cells in an aged microenvironment.

**Figure 4:**
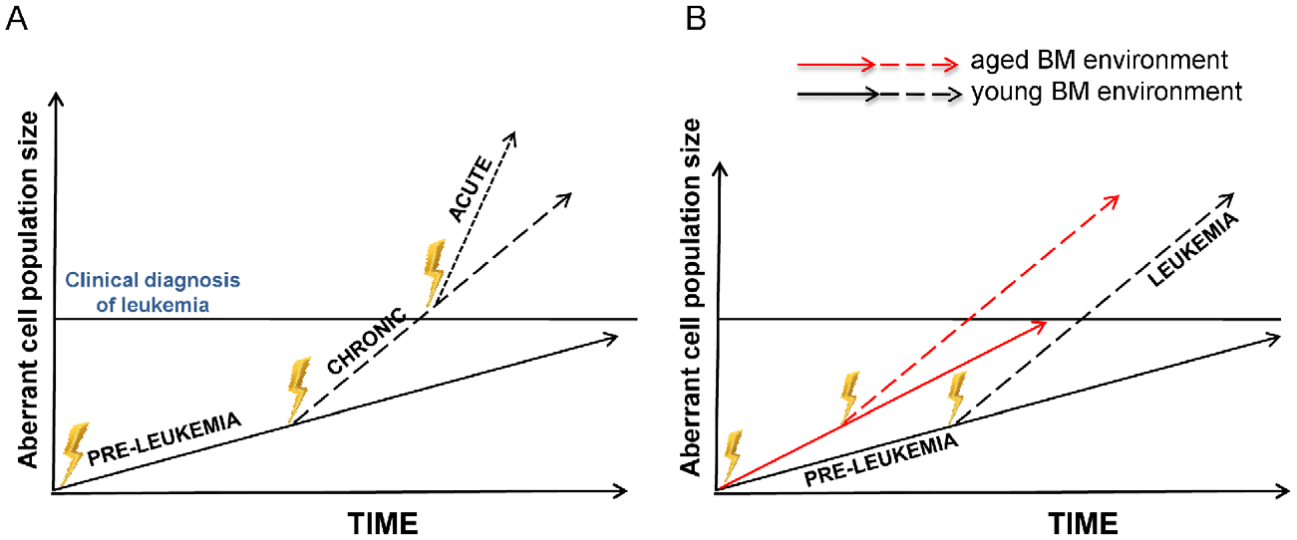
Model for the influence of an aged microenvironment on leukemia progression. (**A**) The standard model describes leukemia development as the clonal evolution of an aberrant clone: a founder cell mutates through multiple subsequent steps, frequently via a chronic phase (CML) to ultimately result in acute myeloid leukemia (AML). The velocity of expansion of the aberrant clone increase along the three phases of leukemia (pre-leukemia, chronic leukemia, acute leukemia). The transitions between the phases are not well described in both cellular and molecular terms, but might be caused by intrinsic/genetic and/or extrinsic changes (figure adapted from ref. [46]). (**B**) An aged microenvironment increases the velocity of the expansion of an aberrant pre-leukemic clone. The faster expansion of myeloproliferation-initiating stem cells in aged BM thus promoting the leukemic process. In a larger aberrant cell population, the probability of generating additional hits is increased, resulting in a more likely and thus earlier transition to the next phase of leukemia.

The widely accepted clonal evolution model of cancer states that accumulation of mutations in aberrant cells lead to a dominant clone that will subsequently acquire additional mutations [3], [40], [41]. Selection of the most competitive mutant clone will ultimately result in tumor formation (Figure 4A) [42]. Based on our data we propose a novel modified clonal evolution model in which an aged BM niche/microenvironment exerts a selection pressure which is distinct from a young microenvironment. Our data with respect to AML-ETO driven AML suggests that the aged niche does not directly accelerate the disease-transition to a chronic phase – it rather accelerates the expansion of the aberrant dominant LIC clone (Figure 4B). This results in a steeper slope for the expansion of the aberrant clone in an old compared to a young niche. A larger aberrant cell population might then increase the probability towards a fully malignant transition, in which case the time of transition to acute phase would be faster in an aged microenvironment. Consistent with this interpretation of our data are for example findings showing that blast cell numbers [43] or a high number of CD34+ in the BM of AML patients [44], [45] correlated with shorter survival, supporting a relationship between higher leukemia-initiating cell burden and poor prognosis.

In summary we suggest that aging of the niche/microenvironment plays a role in aging-associated increased incidence of leukemia by expanding leukemia-initiating cells. Further studies though are warranted to fully elucidate cellular and molecular mechanisms of aging of the microenvironment and increased incidence of aging-associated leukemia.

## Materials and Methods

### Retroviral Particles Production

Murine stem cell virus (MSCV) IRES/GFP was used as a control vector/virus. The AML1-ETO IRES/GFP vector was already described (ref. [31]). Cell-free supernatants containing retroviral particles were generated by transient transfection of Phoenix-gp packaging cells using Calcium Phosphate Transfection kit (Invitrogen, Carlsbad, USA). The collected supernatants were tittered on 3T3 cell line (purchased from American Type Culture Collection, ATCC number: CRL-1658) and the high virus titer fractions (1.3–3.2×10^6^) were used for BM cell transduction.

### Animals

Young (2–4 months old) and aged (18–23 months old) C57BL/6 mice were obtained from in house colonies or purchased from Janvier (St. Berthevin Cedex, France).

Ethics Statement: Animal experiments were carried out in University Ulm accordance with Tierschutzgesetz Paragraph 8 Abs. 1 and 3, were approved by the “Regierungspräsidium Tübingen” (Az:35/9185.81 protocol number 957). The study was performed in cooperation with the Comprehensive Mouse and Cancer Core in the Division of Experimental Hematology, Cincinnati Children's Hospital Medical Center in accordance with the approved animal handling protocols (Nr. 9D04039 and 8D10089) provided by the Institutional Animal Care and Use Committee (IACUC) Cincinnati.

### Retroviral Gene Transfer and Bone Marrow Transplantation

Femora and tibiae were isolated from 2–4 month old C57BL6 mice 3 days post injection of 5-FU (135 mg/kg of body weight, i.p.). BM was flushed from the long bones and mononuclear cells were isolated by low-density centrifugation (Histopaque 1083, Sigma). Cells were pre-stimulated in 6 well non-tissue culture dishes for 2 days in IMDM medium (Lonza, Verviers, Belgium)/10%FBS (HyClone, Thermo Scientific, South Logan, Utah) containing 100 ng/mL mSCF, TPO, G-CSF (Prospec, Tany TechnoGene Ltd., Rehovot, Israel), 1% penicillin/streptomycin, and 2 mM glutamine at a density of 1.7–2×10^7^ cell/well. The transduction was performed on day 3 in six-well plates on RetroNectin-coated (9.5 µg/cm^2^; TaKaRa, Otsu, Japan) non-tissue culture dishes. Virus preloading was carried out with cell free virus supernatant by centrifugation (40 min., at 4 C, 1300 *g*). The pre-stimulated BM cells were seeded on top (1 ml of 1.2–1.8×10^6^ prestimulated cells/well) with fresh thawed viral supernatant (2 ml) supplemented with cytokines (100 ng/mL mSCF, TPO, G-CSF) with an MOI of 1.4±0.27 among our experiments and incubated overnight. Next morning, the media was changed and the cells were kept for 5 hrs without virus particles. The transduction was repeated with fresh thawed viral supernatant overnight. Next day, the transduced cells were collected with cell dissociation buffer (Gibco) and the transduction efficiency was measured by FACS in small fraction of the samples prior to transplantation. In the 3 independent experiments, the graft contained 3.9%, 12.1%, 12% AML-ETO+ cells and 14.3%, 40.3%, 29.6% GFP+ control transduced cells. In general, the overall transduction efficiency of AML/ETO was inferior to control with respect to total GFP+ cells, but transduction efficiencies at the LSK cell level were similar for both viruses when determined by flow before initiating experiments (control: 67.5%±0.1 GFP+ cells among LSK cells and AML-ETO: 60.6%±11.6 GFP+ cells among LSK cells). Prior to transplantation the young and old recipient mice were irradiated with 9.5 Gy, and cells (approximately 10^6^ cells/recipient) were transplanted into mice by retro-orbital injection. The weight of the animals did not differ significantly among the experimental groups at termination of the experiments.

### Flow Cytometry Analysis of PB, BM and Spleen Samples

Chimerism in peripheral blood (PB) was determined in young and aged recipient mice every 4–5 weeks post transplant. PB (retro-orbital bleeding) was incubated with: APC conjugated anti-mouse CD45R/B220 (RA3-6B2) (BD Pharmingen), Alexafluor-700 conjugated anti-mouse CD11b (M1/70) (eBioscience), PE-Cy7 conjugated CD3e (145-2C11) (eBioscinece) and APC-Cy7 conjugated anti-mouse Ly-6G/Ly-6C (Gr-1) (RB6-8C5) (BD Pharmingen) for 20 min on ice. After red blood cell lysis with standard NH_4_CL buffer, cells were analyzed on a BD LSRII flow cytometer. 25–29 week after transplantation 5 old and 8 young control mice and 11 old and 12 young AML-ETO mice were sacrificed and BM and spleen cells were isolated. For stem/progenitor cell measurement, the mononuclear cells (Histopaque 1083, Sigma) were incubated with a cocktail of biotinylated antibodies specific for receptors on differentiated cells: anti-CD11b (clone M1/70), anti-B220 (clone RA3-6B2), anti-CD5 (clone 53-7.3) anti-Gr-1 (clone RB6-8C5), anti-Ter119, anti-CD8a (Clone 53-6.7) (all from BD Pharmingen). Cells were subsequently incubated with anti-Sca-1-PE-Cy7 (clone D7) (eBioscience), anti-C-kit-APC (clone 2B8) (eBioscience), Streptavidin-APC-Cy7 (BD Pharmingen). The EGFP expression was identified within the Lin−, Sca-1+, C-kit+ (LSK) cell population on BD LSRII.

### Endosteal Cell *in Vitro* Co-Culture Assay

To isolate cells close to the endosteum, femora and tibiae were isolated from young (2–3 months old) and aged (22–23 months old) mice. The bones were cleaned and the associated muscle tissues removed. After the BM was flushed, the bones were crushed using scissors and minced with a scalpel in 1.5 mg/ml Collagenase IV (Worthington, Lakewood, NJ, USA)/PBS. The bone chips were further incubated and shaken for 1.5 hrs at 37°C in Collagenase IV/PBS. The bone chips were washed extensively with IMDM/10%FBS and the dissociated cells collected. This endosteal cell fraction was filtered through a 100 µm cell strainer and seeded in 96 well tissue culture plates at a density of 3×10^5^ cells/well in IMDM supplemented with 10% FBS, 1% pen/strep, and 2 mM glutamine. After 2 days of incubation the non-adherent cells were washed off and the adherent cells received 100 ul fresh media. On the next morning cells were used for co-culture experiments. Transduced young hematopoietic cells were seeded in triplicates on top of the adherent young and aged endosteal cells at a cell density of 10^5^ cells/100 ul/well. As control, stroma wells cultured without transduced cells were used. On the 3^rd^ day, the adherent and non-adherent cells were collected using cell-dissociation buffer (Gibco). Both the stroma elements and the hematopoietic cells were isolated together as the LSK contribution from the stroma cell preparation was negligible (less than 2 LSK cells/stroma sample). The samples were stained with biotinylated lineage-marker cocktail and anti-Sca-1-PE-Cy7, anti-C-kit-APC, SA-APC-Cy7. The EGFP expression was identified within the LSK cell population on a BD LSRII. The coculture experiment was repeated three times.

### Statistics

A paired student's t-test was performed to determine the significance of the difference between means of two groups. Values were considered significant when p<0.05.
